# No antibiotics for asymptomatic bacteriuria

**DOI:** 10.1007/s15010-024-02369-9

**Published:** 2024-08-19

**Authors:** Annika P. Schnell

**Affiliations:** Friedrich-Alexander-Universität Erlangen-Nürnberg (FAU), 91054 Erlangen, Germany

## Image


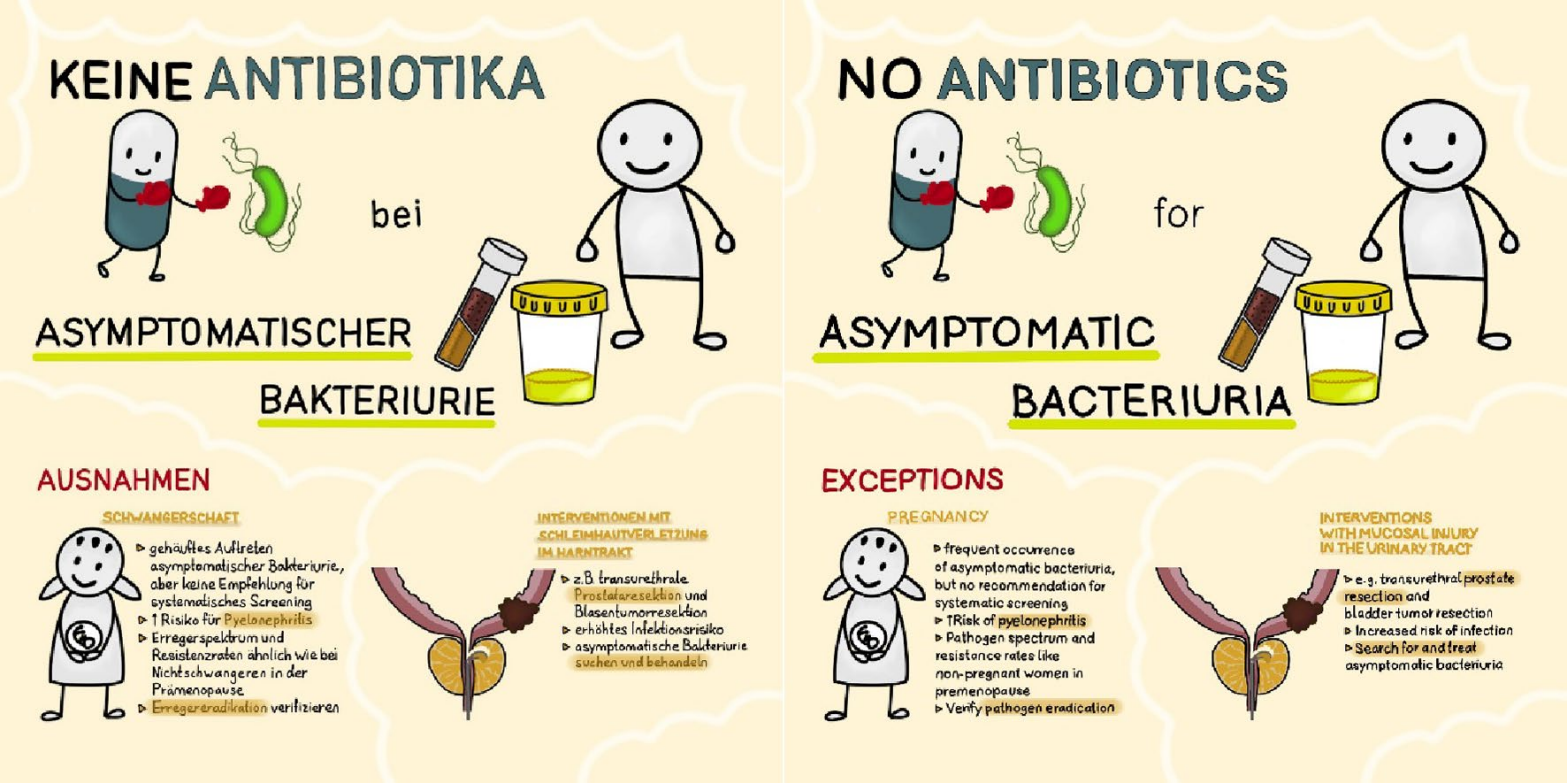


## Image description

“Choosing wisely” (Klug entscheiden) is an initiative of the German Society of Internal Medicine (Deutsche Gesellschaft für Innere Medizin e.V., DGIM) in cooperation with other medical societies, targeting the overuse and underuse of diagnostics and therapeutics. To this end, it offers recommendations for diagnosis and treatment options that are optimal for patients to promote indication quality in medicine [1–4].

A highly topical issue in infectious disease is the use of antibiotics and other anti-infectives. Overuse promotes antimicrobial resistance, a substantial threat faced by healthcare systems worldwide. On the one hand, rational use of antibiotics is beneficial for the individual patient. On the other hand, it can help reduce the spread of resistant pathogens [4].

One of the recommendations is “Do not treat asymptomatic bacteriuria with antibiotics” [1, 3]. The accompanying images illustrate this recommendation for both doctors and patients. It was created as part of a creative competition celebrating the 50th anniversary of the journal INFECTION and was awarded third place.

Earlier communications regarding the “choosing wisely” initiative are listed in the references.

## Data Availability

No datasets were generated or analysed during the current study.
